# Low Economic Class Might Predispose Children under Five Years of Age to Stunting in Ethiopia: Updates of Systematic Review and Meta-Analysis

**DOI:** 10.1155/2020/2169847

**Published:** 2020-12-12

**Authors:** Mesfin Wudu Kassaw, Aschalew Afework Bitew, Alemayehu Digssie Gebremariam, Netsanet Fentahun, Murat Açık, Tadesse Awoke Ayele

**Affiliations:** ^1^Department of Nursing, College of Health Science, Woldia University, Woldia, Ethiopia; ^2^Department of Public Health, College of Health Science, GAMBY Medical College, Bahir Dar, Ethiopia; ^3^Department of Public Health, College of Health Science, Debre Tabore University, Debre Tabore, Ethiopia; ^4^Department of Nutrition and Dietetics, College of Medicine and Health Sciences, Bahir Dar University, Bahir Dar, Ethiopia; ^**5**^ Department of Nutrition and Dietetics, Faculty of Health Sciences, Ankara University, Ankara, Turkey; ^6^Department of Public Health, College of Health Science, University of Gondar, Gondar, Ethiopia

## Abstract

**Background:**

Malnutrition is major public health problem worldwide, particularly in developing countries including Ethiopia. In 2016, out of 667 million children under five years of age, 159 million were stunted worldwide. The prevalence of stunting has been decreasing greatly from 58% in 2000 to 44% in 2011 and 38% in 2016 in Ethiopia. However, the prevalence of stunting is still high and considered as public health problem for the country. The aim of this systematic review and meta-analysis is to assess the prevalence of stunting and its associations with wealth index among children under five years of age in Ethiopia. *Methodology*. The databases searched were MEDLINE, Scopus, HINARI, and grey literature studies. The studies' qualities were assessed by two reviewers independently, and any controversy was handled by other reviewers using the Joanna Briggs Institute (JBI) critical appraisal checklist. The JBI checklist was used in assessing the risk of bias and method of measurement for both outcome and independent variables. Especially, the study design, study participants, definition of stunting, statistical methods used to identify the associations, results/data presentations, and odds ratios (ORs) with confidence intervals (CIs) were assessed. In the statistical analysis, the funnel plot, Egger's test, and Begg's test were used to assess publication bias. The *I*^2^ statistic, forest plot, and Cochran's *Q*-test were used to deal with heterogeneity.

**Results:**

In this review, 35 studies were included to assess the pooled prevalence of stunting. Similarly, 16 studies were used to assess the estimated effect sizes of wealth index on stunting. In this meta-analysis, the pooled prevalence of stunting was 41.5% among children under five years of age, despite its considerable heterogeneity (*I*^2^ = 97.6%, *p* < 0.001, *Q* = 1461.93). However, no publication bias was detected (Egger's test *p*=0.26 and Begg's test *p*=0.87). Children from households with a medium or low/poor wealth index had higher odds of stunting (AOR: 1.33, 95% CI 1.07, 1.65 or AOR: 1.92, 95% CI 1.46, 2.54, respectively) compared to children from households with a high/rich wealth index. Both of the estimated effect sizes of low and medium wealth indexes had substantial heterogeneity (*I*^2^ = 63.8%, *p* < 0.001, *Q* = 44.21 and *I*^2^ = 78.3%, *p* < 0.001, *Q* = 73.73) respectively). In estimating the effect, there was no publication bias (small-studies effect) (Egger and Begg's test, *p* > 0.05).

**Conclusions:**

The pooled prevalence of stunting was great. In the subgroup analysis, the Amhara region had the highest prevalence of stunting, followed by the Oromia and Tigray regions, respectively. Low economic status was associated with stunting in Ethiopia. This relationship was found to be statistically more accurate in Oromia and Amhara regions. The government should emphasize community-based nutrition programs by scaling up more in these regions, just like the Seqota Declaration.

## 1. Background

This is an updated systematic review and meta-analysis version which conducted with adding papers that were not included in the previous two systematic reviews and meta-analysis on the title [1, 2]. In addition, the current systematic review and meta-analysis allow omitting of papers that were included in the previous two systematic reviews and meta-analysis but conducted before a long time [1, 2]. The culture, and socioeconomic status of the Ethiopian community were changing over time, particularly, 2010 was the time in which the Ethiopian economy starts to grow radically [3]. In addition, 2010 was the beginning of the Growth and Transformation Plan I (GTP I) of Ethiopia [4].

Because of such reasons, authors do not allow papers that conducted before 2010. In contrary, the ending period to include studies was 2019, which was the ending period for GTP II [5]. Thus, the aim of this systematic review and meta-analysis was to measure the pooled prevalence of studying during the GTP 1 (2010–2015) [4] and GTP II (2015–2019) [5] of the country. Although the major reasons to update the review are stated, the previous two reviews were also had limitations that might affect the conclusion of their findings. One of the previous systematic reviews conducted by Ahmed et al. [1] focused on pooled prevalence of stunting, wasting, underweight, and its determinants. The other systematic review was conducted by Kalkidan and Tefera that reported determinates of stunting, wasting, and underweight [2]. Both of these systematic reviews and meta-analysis considered more than 3 outcome variables, which makes the data handling, searching strategy, and data analysis more complex. The relative stability of stunting [6] independent of the improvement of food consumption in Ethiopia [7] is the other reason to update the title despite the presence of these two systematic reviews with limitations. Despite the description to update the former systematic review, malnutrition is remained as a major public health problem in developing countries, including Ethiopia, all over the world [8]. Unfortunately, children and women are the most vulnerable population for malnutrition globally [9], and in Ethiopia [7]. In 2010, globally stunting affected approximately 162 million children under the age of 5 years [10]. In 2016, out of 667 million children, 159 million were stunted and 50 million were weakened all over the world [11]. Together with its high prevalence, malnutrition causes physical and mental developmental retardation and drop off the economic growth [12]. Worldwide, a huge gross domestic product (GDP) is lost annually due to stunting alone [13]. Stunting has also long-term effects on individual and societal health which includes diminished cognitive development, reduced productive capacity, poor health, and increased risk of degenerative diseases [14, 15]. The prevalence of stunting reaches its peak between 12 and 24 months of childhood age (40%–54%), continues to increase until 36 months of age (58%), and then remains fairly stable until 5 years of age (55%) [16]. According to the World Health Organization (WHO) report, the prevalence of stunting decreased between 2000 and 2016 [17, 18]. The relative stability of stunting, its high prevalence, its severe complications, and chronic clinical features are the reasons to select it for systematic review over wasting or underweight which are usually the acute types of malnutrition with treatable complications [19].

Of the repeatedly reported factors of stunting, inadequate nutrition and repeated occurrence of infection during the first 1000 days of a child's life were the major determinants [20–24]. Birth weight, birth interval, maternal occupation, residence, food security, source of drinking water, dietary diversity, vitamin-A supplementation, type of feeding, duration of exclusive breastfeeding, wealth index, and time of initiation of complementary feeding also associated with stunting [21–34] in global perspectives. In Ethiopia, duration of breastfeeding, maternal working status, maternal education, vitamin-A supplementation, family size, monthly income, paternal education, birth weight, antinatal care (ANC) visits, maternal height, maternal body mass index (BMI), birth order, and dietary diversity associated with stunting [35–43]. Thus, updating the pooled prevalence of stunting and estimating the effect of wealth index on stunting is crucial to formulate interventions.

## 2. Objective

The objective of this systematic review is to assess the pooled prevalence of stunting and measure the estimated effect of wealth index on stunting among children under five years of age in Ethiopia.

## 3. Methods

### 3.1. PROSPERO Registration

The protocol had been registered in the PROSPERO (registration number CRD42019127005). PROSPERO is an international database of prospectively registered systematic reviews in health and social care. Key features from the review protocol are recorded and maintained as a permanent record. Systematic reviews should be registered at inception to help avoid unplanned duplication and to enable comparison of reported review methods with what was planned in the protocol.

### 3.2. Searching Strategies

We conducted a systematic and exhaustive search of literature studies published between 2010 and 2019. We assumed that the socioeconomic status of the country in the past 10 years might have not significant differences. Thus, the articles that published in the past 10 years might be homogeneous and provide more precise data against the current socioeconomic status of the nations. Designing comprehensive search strategy was a priori, in which the search term was developed using Boolean operators and adapted to databases. The databases or online libraries used were MEDLINE, Scopus, HINARI, Google Scholar, and Google. The searches for unpublished studies were done from institutional libraries and research gate. All searches were limited to the English language and publication dates between 01 January 2010 and 15 November 2019. The searching strategy used in MEDLINE was composed of (((((((((((((Child) OR infant) OR children) OR preschool children) OR under five years children) AND wealth index) OR wealth quintiles) OR economic status) AND stunting) OR malnutrition) OR nutritional status) OR growth failure) OR chronic malnutrition) AND Ethiopia).

## 4. Inclusion and Exclusion Criteria

### 4.1. Inclusion Criteria

Observational studies (cross-sectional, analytical cross-sectional, case-control, cohort, and comparative observational) with one of the following criteria were included:

- Studies done on under-five-year-old children
- Articles published only using the English language
- Studies that published between 01 January 2010 and 15 November 2019 and reported a stunting prevalence or wealth index as factors of stunting
- Studies that reported two prevalence values or odds ratios because of their nature of design were also included

### 4.2. Exclusion Criteria

However, studies with one of the following criteria were excluded:

- Studies using nonobservational study design
- Articles with no full text or data that were difficult to extract the odds ratio, despite contacting the corresponding author(s)
- Studies carried out in healthcare facilities, as illness greatly affected the nutritional status of the study participants
- Studies with methodological limitations, such as incorrect outcome ascertainment criteria
- Studies that measured the outcome variable (stunting) other than Z-score

### 4.3. Measurement of the Outcome Variable and Study Variables

Since stunting is a century's burden of Ethiopia and has serious complications, we prefer it over wasting or underweight. Stunting is defined as having a height-for-age Z-score (HAZ) < -2 SDs for a child's age and gender. Stunting is the impaired growth and development that children experience from poor nutrition, repeated infection, and inadequate psychosocial stimulation. It is classified as a HAZ < -2SDs, a measure based on comparisons of a child's height (cm) and age (months) to WHO standards [44]. Wealth index is a composite measure of a household's living standard that is separated into five quintiles.

It is calculated using the ownership of a household's selected assets (rural or urban), such as televisions and bicycles, materials used for housing construction, types of water access, and sanitation facilities [45]. In this review, the wealth index was categorized into three quintiles (low/poor, medium, and high/rich) from the primary studies that reported the wealth index using five categorized quintiles: poorest, poorer, medium, richer, and richest as per the EDHS recommendation [45]. The poorest and poorer wealth indices were grouped into low/poor, medium wealth index into medium, and the richer and richest wealth indices into high/rich.

### 4.4. Study Selection and Data Collection

The studies identified through the database searches were combined, exported, and managed using Endnote version X9.2 software (Thomson Reuters, Philadelphia, PA, USA). Duplicated studies were removed, and the full texts of the articles were searched by Endnote software. Three reviewers (AA, AD, and MW) assessed the studies for eligibility independently. These authors are senior lectures and took a systematic review and meta-analysis training for one year. Two of the authors (AD and MW) have more than 10 publications including systematic review and meta-analysis articles. The reviewers assessed papers starting with the title, abstract, and then full text. The discrepancies between these three reviewers were solved by discussion with other reviewers (TA, MA, and NF). These three authors were senior researchers. Two of the authors (TA and NF) have more than 50 publications including systematic review and meta-analysis. Also, they are trainers of systematic reviews and meta-analysis.

### 4.5. Quality Assessment of Individual Studies

The JBI checklist was used in assessing the risk of bias and method of measurement for both outcome and independent variables. Especially, the study design, study participants, definition of stunting, statistical methods used to identify the associations, results/data presentations, and odds ratios (ORs) with confidence intervals (CIs) were assessed. The retrieved studies were assessed based on their designs using the JBI checklist for cross-sectional studies [46], case-control studies [47], and cohort studies [47]. With regard to the critical appraisal process, studies that got 5 out of 8 for cross-sectional, 6 out of 11 for cohort, and 5 out of 10 for case control were considered to have good quality/low risk. Studies that have scores below the cut points in the JBI checklist were considered as poor quality/high risk. All the authors were involved in appraising the quality of articles. A single article was assessed by two authors, and when there was controversy, a third author reviewed the paper.

The teams arranged for the critical appraisal were MW & AA, TA & NF, and AD & MA. However, when the disagreement happened, any of these authors from any of the team was assigned. The decision made based on the evaluation of the third author. If they rejected the article, they also mentioned the reason of rejection. In addition, the existence of publication bias was assessed using funnel plots and symmetry. Egger's test was also computed [48] to confirm the subjective assessment. In Egger's test, a *p* value of <0.05 was used to declare the presence of a statistical significance publication bias.

### 4.6. Data Extraction and Management

The data extraction sheet was piloted using nine papers that were selected randomly. Findings of the prevalence section (P) and its associations with wealth index (OR) from each study were summarized by all authors using Microsoft Excel. The teams of authors to extract the data were MW & AA, TA & NF, and AD & MA. Finally, the extracted Excel data were merged together. When there is a discrepancy between the two authors within the team, a third author was assigned and managed the disagreement. Any mistyping of data and other concerns were resolved through crosschecking with the included papers. The data extraction format was prepared by all the authors through the assistance of the Joanna Briggs Institute (JBI) data extraction tool for prevalence and incidence and associations studies. From each study, the authors, year of publication, study design, sample size, outcome, anthropometric analysis, prevalence of stunting and its standard error, and wealth index estimates with its standard error were extracted. Electronic mails were sent to the corresponding or first authors of the studies or abstracts for missing information, and we waited 3 to 4 weeks for their responses. When there were no responses, the papers were excluded with the reason that the papers were not available.

### 4.7. Statistical Analysis

The extracted data were exported to STATA/SE version 14.0 software for analysis. The pooled prevalence of stunting and its associations with wealth index were determined by the random effect model using Der-Simonian-Laird weighting [49]. Since the studies retrieved were heterogeneous by study area, sample size, design, population, and study period, we decided to use the random effect model. Our statement is also concurrent with evidence that heterogeneity in meta-analysis is mostly known to be inevitable due to differences in study quality, sample size, method. and different outcomes measured across studies [50, 51]. The statistical heterogeneity was checked by forest plot subjectively and Cochran's *Q*-test and *I*^2^ statistic [52] objectively.

In order to minimize the variance of point estimates between primary studies, a subgroup analysis was carried out. When the heterogeneity was consistent, sensitivity analysis was used to determine the effect of each study on the pooled prevalence. The presence of publication bias (small study effect) was also checked using graphical tests (funnel plots) and the objective tests, Egger's test [48], and Begg's test. Both Egger's and Begg's tests were statistically significant (*p* < 0.05), indicating the presence of a small study effects. Whenever there was publication bias, it was handled by nonparametric trim and fill analysis using the random effect model [53].

## 5. Results

### 5.1. Literature Searches and Selections

**Figure 1:**
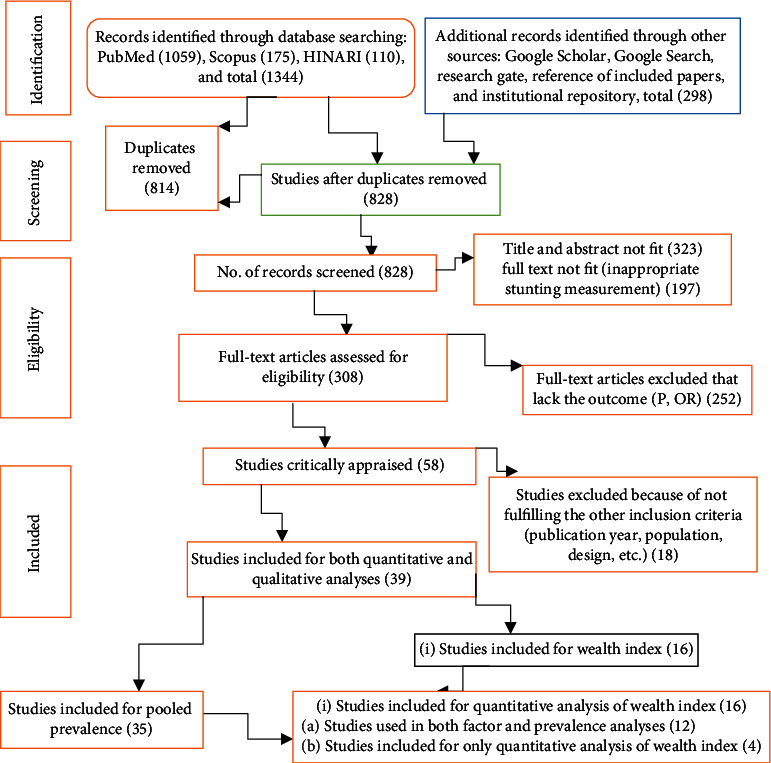
The PRISMA flowchart that indicates study selection and appraisal process.

In the initial search, 1642 records were found from the electronic databases. The databases searched and their records found from were MEDLINE (1059), Scopus (175), Google Scholar (236), HINARI (110), and grey literature studies [54, 55]. The grey literature studies considered in this review were Google Search, Research gate, Google Scholar, and institutional repository. Of the 1642 papers, only 828 papers remained for further evaluation after removing duplicates. Upon the appraisal of the titles and abstracts, 323 records were excluded. There were 58 full-text articles that were eligible for critical appraisal. However, 18 records were further excluded for not fulfilling the inclusion criteria. Finally, 39 articles remained for this review and meta-analysis [25, 26, 42, 55–90]. Of these 39 studies, 35 of the studies were used in assessing the pooled prevalence of stunting [25, 26, 42, 55–62, 64, 65, 67–81, 83, 84, 86, 88–91], and 16 of the studies were used to estimate the effect of wealth index on stunting [55–57, 65, 70, 73, 76, 77, 79, 80, 82, 83, 85–88] (Figure 1). Of the 16 papers that used to estimate the effect of wealth index on stunting, four were not used in calculating the pooled prevalence of stunting [82, 83, 85, 87].

### 5.2. Characteristics of the Studies

The pooled prevalence of stunting was computed using 35 studies that gave 51,452 children aged birth to 5 years old. The sample size of those primary studies ranged from 214 to 9893 under-five children. Of the sample children for the review, 24,107 had the outcome of stunting, or and wealth index [25, 26, 42, 55–62, 64, 65, 67–81, 83, 84, 86, 88–91]. Only one study was conducted at a health facility [25], whereas the remaining 34 studies were conducted at the household level. All the studies considered in the pooled prevalence of stunting were studied using cross-sectional design. Eight of the papers were published from 2010 to 2014 [61, 65–67, 73, 76, 77, 79], and the remaining 31 papers were published from 2015 to 2019. Three of the studies were conducted from Ethiopian demography health and survey (EDHS) data [62, 85, 91], and two of the papers were studied using secondary data [25, 61]. Five of the studies considered children aged 6–24 months [56, 57, 67, 70, 82], and one study considered children aged 24 to 59 months old [59]. However, all the other studies were conducted on children aged 6 to 59 months old. Nine studies were from Amhara [26, 42, 65, 76, 79, 80, 84, 86, 89] and SNNP regions [55, 58, 60, 67, 73, 75, 78, 82, 83]. Studies from Oromia Region (*n* = 7) [25, 56, 59, 66, 71, 77, 87], Tigray Region(*n* = 3) [64, 69, 70], Somalia Region, Ethiopia (*n* = 2) [57, 74], Benishangul-Gumz Region (*n* = 1) [88], and Afar Region (*n* = 2) [68, 81], and six studies were nation-based [61, 62, 72, 85, 90, 91] (Table 1).

Three of the studies did not report the sex of the study population, children [66, 67, 86], but all the other studies reported the sex of children. Accordingly, boys contributed to 65.2% (33,533) of the study population (Table 1).

Most papers were published in a peer-reviewed journal. However, only one study [88], and one mini demography and health survey (EDHS) report [90] fulfilled the criteria and included in this systematic review and meta-analysis. Regarding the associations between wealth index and stunting, the sample size of the studies was 22,183 children aged birth to 5 years. With regard to the effect estimation of the associations, the sample size ranged from 214 to 7452 participants. All the studies that used to estimate the effect sizes of wealth index on stunting were conducted at household level [55–57, 65, 70, 73, 76, 77, 79, 80, 82, 83, 85–88]. Considering this associations between stunting and wealth index, one of the papers studied used a cohort design [87], two of the papers studied used case-control design [70, 82], and the remaining 13 studies studied used cross-sectional design. Six of the studies [65, 76, 79, 80, 82, 86] were from Amhara Region, three were from South Nations, Nationalities, and Peoples' Region (SNNPR) [55, 73, 83], three were from Oromia Region [56, 77, 87], one was from Tigray Region [70], one was from Somalia Region [57], and one was from Benishangul-Gumuz Region [88], while one study was a nation-based study (Ethiopia) [85] (Tables 1 and 2).

More important descriptions for this review came from a study by Behailu et al. that used a comparative cross-sectional design and reported two prevalence values and two odds ratios (OR). Thus, we considered this paper as two papers in the meta-analysis section, but it was cited only once. Therefore, the data on the pooled magnitude of stunting were generated from 36 [76] studies, and the pooled estimate of the wealth index was produced using 17 studies [76], but the number of citations was indicated as 35 and 16 for the pooled prevalence and effect estimate, respectively. Therefore, all the forest plots have one additional paper than the papers mentioned in the PRISMA chart.

### 5.3. Systematic Review

**Figure 2:**
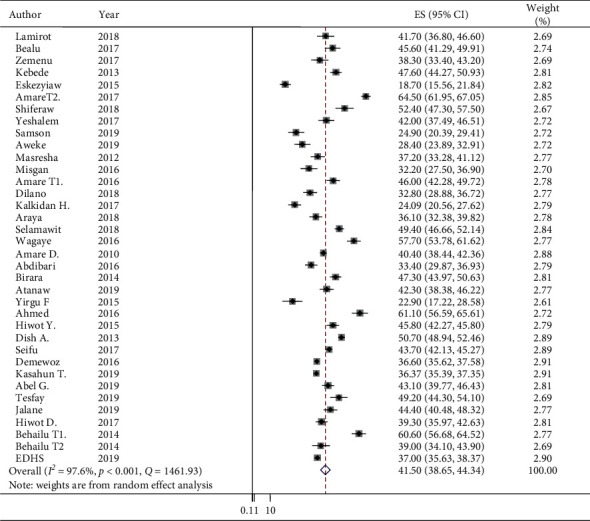
The prevalence of stunting among children under five years of age and its 95% CI in Ethiopia, 2010–2019.

The prevalence of stunting from each primary studies varied from 18.7% to 64.5% (Figure 2).

Most of the studies (33 studies) included were from seven regions of Ethiopia, and 6 were country-based studies [61, 62, 72, 85, 90, 91]. The highest numbers of studies were reported from Amhara Region, nine of the prevalence studies [26, 42, 65, 76, 79, 80, 84, 86, 89], and six of the wealth index studies [65, 76, 79, 80, 82, 86], while the lowest number of studies were from Benishangul-Gumuz Region, only one study was included in the prevalence section [88]. Considering the associations between the wealth index and stunting, there was one study for each region, including Tigray Region, Ethiopia [70], Somalia Region, Ethiopia [57], and Benishangul-Gumz Region, Ethiopia [88], and there was one nation-based study (country-wide) [85]. The highest prevalence of stunting was reported from Amhara Region (64.5% [80] and 60.6% [76], followed by the Oromia region (61.1%) [59], whereas the lowest prevalence was from the SNNP region (18.7%) [78], followed by the Somalia region (22.9%) [57] (Table 1). The highest odds of stunting because of low economic class were reported from Tigray (AOR 6.0) [70] and Oromia (AOR 4.5 and 3.3) [56, 77]. Similarly, the highest odds of stunting because of having a medium economic class were from SNNP (AOR 2.5) [83], Tigray (AOR 2.4) [70], and Oromia (AOR 2.3) [77].

### 5.4. Meta-Analysis

Thirty-five studies were included to assess the pooled prevalence of stunting [25, 26, 42, 55–62, 64, 65, 67–81, 83, 84, 86, 88–91]. On the other hand, 16 studies were used to estimate the pooled effect sizes of wealth index on stunting [55–57, 65, 70, 73, 76, 77, 79, 80, 82, 83, 85–88]. The procedure we followed while including, excluding, appraising, and extracting papers was presented in the Preferred Reporting Items for Systematic Reviews and Meta-Analyses (PRISMA) flowchart (Figure 1) [92].

### 5.5. Prevalence of Stunting in Ethiopia

The pooled prevalence of stunting in Ethiopia was 41.5% (95% CI: 38.65, 44.34), despite a considerable heterogeneity (*I*^2^ = 97.6% and *p* < 0.001). Cochran's Q-test and *I*^2^ statistics, as well as forest plot and Galbraith plot, were considered to deal with this high degree of heterogeneity. The Galbraith plot indicated that more than 26 of the points or studies were outside of the 95% CI, and the CIs were not overlapping on the forest plot (Figure 2).

### 5.6. Heterogeneity Deal

The heterogeneity among studies in assessing prevalence among 35 studies by region was quite high. The *I*^2^ statistics varied from 89.4% at Somalia Region to 98.6% at the country-based studies. The prevalence of stunting (from the lowest to the highest magnitude of stunting) was 28.4% in Somalia Region, 32.8% (single study prevalence) in Benishangul-Gumuz Region, 36.4% in South Nations, Nationalities, and Peoples' Region (SNNPR) of Ethiopia, 37.7% in Afar Region, 40.1% at the country-based study (Ethiopia), 42.5% in Tigray Region, 43.5% in Oromia Region, and 48.2% in Amhara Region, with considerable high heterogeneity. The heterogeneity of the prevalence estimates among the subgroups of 35 studies on stunting by population of the study was also very high. The *I*^2^ statistics for children ≤2 years old (6 to 24 months) was 93.0%, while it was 97.6% for children less than 5 years old (6 to 59 months old). The prevalence of stunting among children ≤2 years old (6 to 24 months) was 28.16% (95% CI: 18.83, 37.48), while it was 42.68% (95% CI: 39.78, 45.59) among children <5 years old.

### 5.7. Sensitivity

Sensitivity analysis was performed using 26 studies by removing data from the meta-analytic model in order to examine the influence of studies with low quality or high bias on the pooled prevalence of stunting. After 10 studies removed due to being highly biased, the prevalence became 43.19% (95% CI: 42.62, 43.76, *I*^2^ = 97.3%, and Cochran's *Q* = 927.85). This sensitivity analysis prevalence was put within the 95% CI of the pooled magnitude of stunting (41.5%) (95% CI: 38.65, 44.34, *I*^2^ = 97.6%, and Cochran's *Q* = 1461.93). Thus, the sensitivity analysis assured that quality of studies did not significantly affect the pooled random prevalence of stunting (Supplementary Figure 1).

### 5.8. Cumulative Meta-Analysis

**Figure 3:**
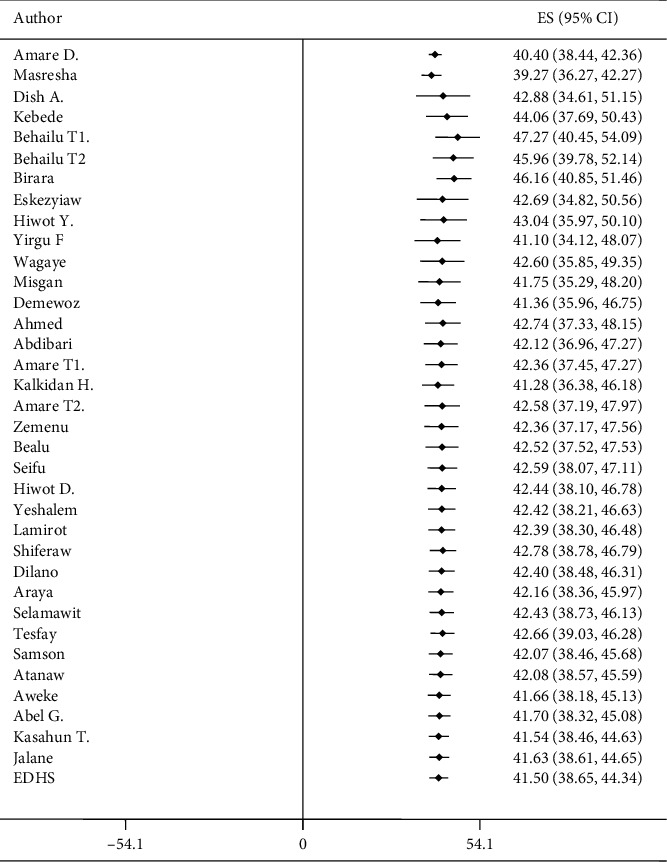
Trends of stunting from 2010 to 2019 using cumulative meta-analysis in Ethiopia.

The cumulative meta-analyses indicated a stabilized trend of stunting prevalence among under-five children in the last 10 years, 2010 to 2019. The prevalence of stunting in 2010 and 2012 was lower than studies reported more recently from 2016 to 2019. Although the difference was irrelevant, there were upward and downward trends of stunting in the last 10 years. The prevalence of stunting was downward for the period 2010–2012, 2014–2015, and 2015–2016. However, the trend of stunting from late 2016 to 2019 was standing at 41% and 42% in down and up trends, with a slight difference in each year. For all years, a significant upward trend of stunting occurred in the period from 2012 to 2014 (Figure 3).

### 5.9. Publication Bias

The publication biases in this meta-analysis were examined using the subjective method, using funnel plot by visual checking for asymmetry, and objectively using Egger's test and Begg's test. In the funnel plot, all studies were distributed symmetrically. Both small- and large-scale studies were distributed on the bottom and top of the graph, assuring the absence of publication bias (Supplementary Figure 2).

The visual inspection of Begg's funnel plot did not identify substantial asymmetry, as nearly all of the studies laid within the 95% CI. Both Egger's and Begg's objective tests also confirmed the absence of publication bias. According to Egger's test, the estimated bias coefficient (intercept) was 2.4, with a standard error of 2.07 and a *p* value of 0.26. Thus, the test provided evidence for the absence of small study effects. Similarly, the *p* value for Begg's test was 0.87 that assured the absence of statistical evidence for publication bias.

### 5.10. Wealth Index and Stunting in Ethiopia

**Figure 4:**
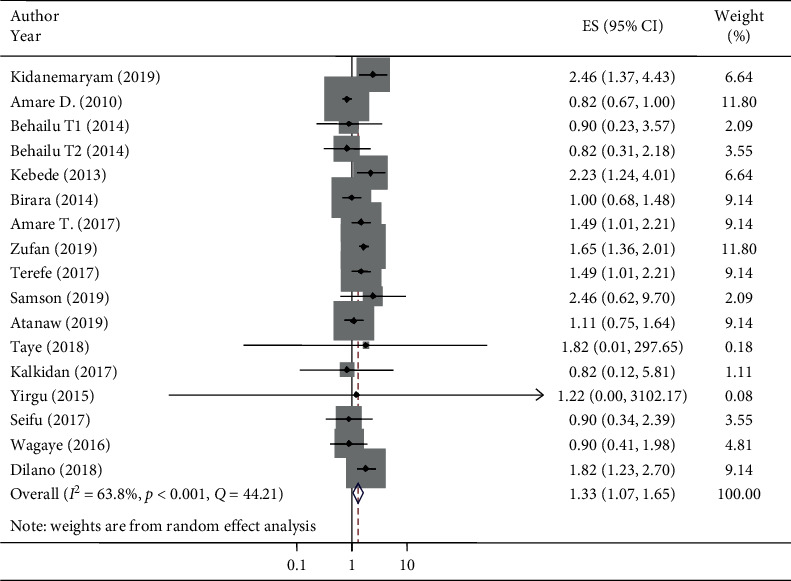
Association of medium household wealth index with stunting among children under five years of age and its 95% CI in Ethiopia, 2010–2019.

**Figure 5:**
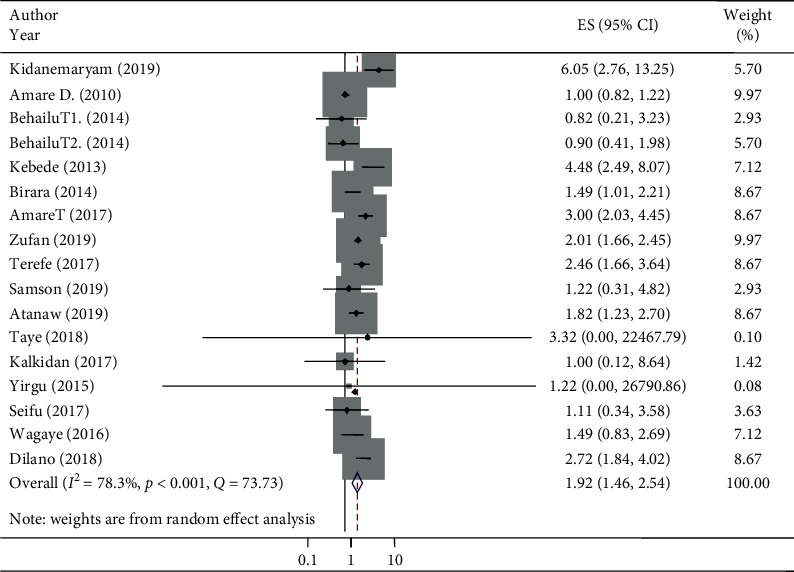
The pooled associations of stunting and poor household wealth index in Ethiopia.

Sixteen studies were included to estimate the associations between the wealth index and stunting. The AOR of stunting varied from 0.83 [76] to 2.46 [70] from medium wealth index households and from 0.83 [76] to 6.05 [70] from low/poor wealth index households as the primary studies indicated. The AOR assured that the wealth index of households was associated with the prevalence of stunting in under-five children in Ethiopia from studies conducted between 20 January 2010 and 15 November 2019 [80, 82, 86]. In this meta-analysis, the odds of stunting increased at medium wealth index households compared to high/rich wealth index households (AOR 1.33, 95% CI: 1.07, 1.65) (Figure 4). Similarly, the odds of stunting at low/poor wealth index households were greater compared with high/rich wealth index households, which was associated with stunting (AOR 1.92, 95% CI: 1.46, 2.54) (Figure 5). The heterogeneity of pooled random effect size estimates among the 17 AOR reports using 16 studies on stunting and associations with low/poor or medium wealth index households was substantial (*I*^2^ = 63.8% and 78.3% and *p* < 0.001 for both low/poor and medium wealth index households, respectively) [52]. In addition to Cochran's *Q*-test and *I*^2^ statistic, both the forest plot and Galbraith plot were considered to deal with this substantial degree of heterogeneity for both low/poor and medium wealth index households against the high/rich wealth index households. The Galbraith plot showed three studies that were out of the 95% CI, and the CIs were not overlapping on the forest plot (Figure 4).

Similarly, in the low/poor wealth index households, the Galbraith plot showed five points were out of the 95% CI, and the CIs were not overlapping on the forest plot (Figure 5).

### 5.11. Heterogeneity Deal

**Figure 6:**
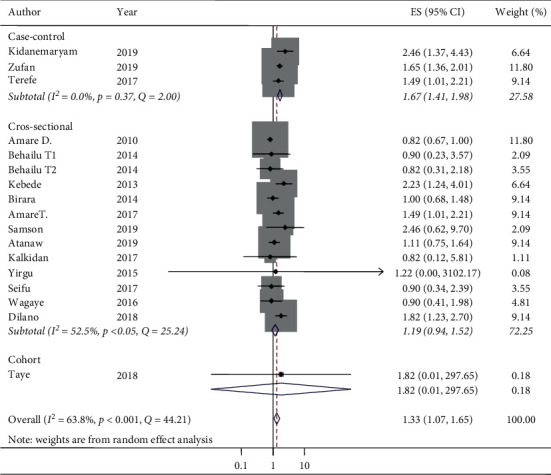
Subgroup analysis (by design) on the association of medium wealth index with stunting and its 95% CI in Ethiopia, 2010–2019.

The pooled *I*^2^ statistic from medium wealth index households and associations with stunting indicated a substantial degree of heterogeneity (*I*^2^ = 63.8%) [52] (Figure 4). The heterogeneity of the pooled random effect size estimates of low/poor wealth index households and associations with stunting had a discrepancy. The pooled *I*^2^statistic from low/poor wealth index households and associations with stunting indicated a considerable degree of heterogeneity (*I*^2^ = 78.3%) [52]. From the subgroup analysis of medium wealth index households and associations with stunting by design, the individual *I*^2^ statistic ranged from 0% in the case-control design to 52.5% in the cross-sectional design, which have a low and moderate degree of heterogeneity, respectively (Figure 6).

**Figure 7:**
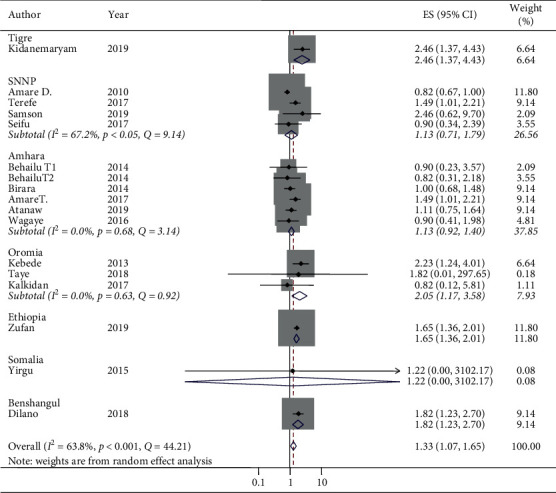
Subgroup analysis (by region) on the association of medium wealth index with stunting and its 95% CI in Ethiopia, 2010–2019.

**Figure 8:**
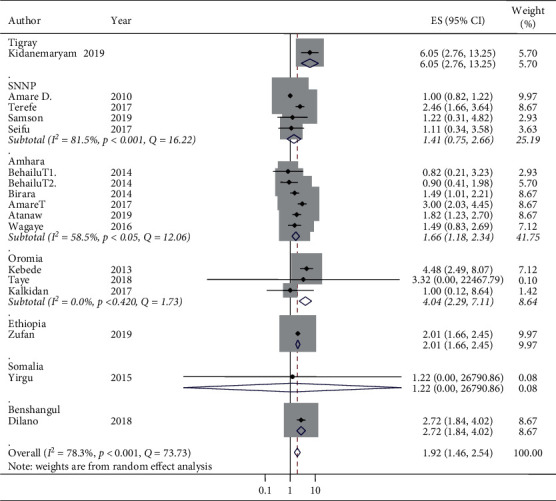
Subgroup analysis (by region) on the association of poor household wealth index and stunting and its 95% CI in Ethiopia, 2010–2019.

**Figure 9:**
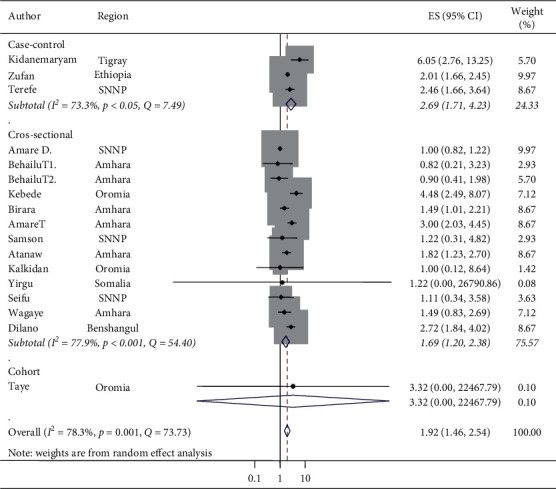
Subgroup analysis (by design) on the association of poor household wealth index and stunting and its 95% CI in Ethiopia, 2010–2019.

The odds of stunting at medium wealth index households relative to high/rich wealth index households in case-control studies were AOR 1.67 (95% CI: 1.41, 1.98) and in cross-sectional studies were AOR 1.19 (95% CI: 0.94, 1.52) (Figure 6). Thus, the subgroup analysis by design in determining the associations between medium wealth index and stunting reported that cross-sectional studies were the more relevant heterogeneity moderators (*I*^2^ = 52.5 and *p*=0.01), but the case-control studies were homogeneous (*I*^2^ = 0% and *p*=0.37). Similarly, there was no statistical associations of stunting and medium wealth index in cross-sectional studies (OR 1.19, 95% CI: 0.94, 1.52), but there was an association between stunting and medium wealth index in case-control studies (OR 1.67, 95% CI: 1.41, 1.98) (Figure 6). The pooled random effect size estimates of medium wealth index and associations with stunting by region had no associations in the two regions. However, in the Oromia region, a significant association was reported. The odds of stunting from medium wealth index households in comparison with high/rich wealth index households were AOR 2.05 (95% CI: 1.17, 3.58) and *I*^2^ = 0%, although the regions considered in the subgroup analysis of medium wealth index and association with stunting were only SNNP, Amhara, and Oromia. The other regions have only a single study and a single AOR was reported in the subgroup analysis of medium wealth index and associations with stunting by region (Figure 7). The pooled random effect size estimates of low/poor wealth index and associations with stunting by region had no association in the SNNP region, but in both Amhara and Oromia regions, a significant associations were reported with a pooled estimate (AOR 1.66 (95% CI: 1.18, 2.34) in the Amhara region and AOR 4.04 (95% CI: 2.29, 7.11) in the Oromia region) (Figure 8). In the subgroup analysis of low/poor wealth index and associations with stunting by design, the individual *I*^2^ statistics ranged from 73.3% in the case-control design to 77.9% in the cross-sectional design, which had a substantial degree of heterogeneity (Figure 9). The odds of stunting from low/poor wealth index households relative to high/rich wealth index households were AOR 2.69, (95% CI: 1.71, 4.23) in case-control studies and AOR 1.69 (95% CI: 1.20, 2.38) in cross-sectional studies (Figure 9). Thus, the subgroup analysis of low/poor wealth index and associations with stunting by design reported that both cross-sectional and case-control studies were relevant heterogeneity moderators (*I*^2^ = 77.9% and 73.3% and *p*=0.01 for both, respectively). Both the case-control and cross-sectional studies had statistically considerable associations with stunting and low/poor wealth index (Figure 9).

### 5.12. Publication Bias

This review assessed the risk of publication bias using funnel plots for symmetry by visual inspection for both the medium and poor household wealth index and associations with stunting. The plot appeared symmetrical and found no publication bias, with most studies concentrated on the top of the plot. The visual inspection of Begg's funnel plot also did not identify substantial asymmetry. Egger's linear regression test revealed evidence of no publication bias (*p*=0.68), and Begg's rank correlation test again assured the absence of publication bias (*p*=0.09).

## 6. Discussion

In this meta-analysis, the pooled prevalence of stunting in Ethiopia was 41.5% (95% CI: 38.65, 44.34). The 2018 united nation UNICEF, WHO, and World Bank joint report indicated that the prevalence of stunting was 22.2% in the world, 9.6% in Latin American and Caribbean countries, 35% in south Asia, and 33.9% in Sub-Saharan Africa [93]. This report confirmed that prevalence of stunting in Ethiopia was higher compared to the world, Latin American and Caribbean countries, south Asia, and Sub-Saharan Africa.

The consistently high prevalence of stunting in Ethiopia after 2010 might be due to the drought that occurred from 2010 to 2014 and the political instability that occurred after 2016. Both the drought and the political instability caused immigration, which may have affected children disproportionately. The prevalence of stunting in our review was greater than another study that reported stunting as 22% globally, 24% in developing countries, and 6% in developed countries [94]. This indicates that stunting in Ethiopia was uniquely high and did not have significant improvements. The UNICEF, WHO, and World Bank joint estimation reported that the international trend in the prevalence of stunting decreased from 39.6% to 23.8% between 1990 and 2014 [95]. However, the prevalence of stunting in Ethiopia is still unchanged from the previous review [1]. The current review showed a decrease of only 0.5% from the previous review, which considers papers from 1997 to 2015, and revealed a 42% prevalence of stunting [1]. This consistently high prevalence of stunting forced us to hypothesizing that achieving the global targets of reducing stunting among children younger than 5 years by 40% in 2030 would be impossible for Ethiopia [96]. This hypothesis agreed with the fourth Ethiopian Health Sector Development Plan. The plan aimed to reduce undernutrition among under-five children by 30% in 2015, but it failed [97]. Beyond this, the current stunting reduction rate in Ethiopia is 2.8%, which is far lower than the expected annual reduction rate of 6% to achieve the WHO's targets of stunting reduction [96]. Therefore, Ethiopia will not reach the United Nations sustainable developmental goals of ending child malnutrition by 2030 or the national commitment to the Seqota Declaration [98] at the current rate of reduction and type of programmes being implemented. On the other hand, this review agreed with the report of the WHO, UNICEF, and World Bank that indicated the number of stunted children in Africa is expected to increase from 56 million in 2010 to 61 million by the year 2025 [99]. The match might be because of analogous geographic location, data, and methodological quality both in the current review and the joint report. Unlike the previous review [1], in this meta-analysis, the prevalence of stunting did not have one directional track, yet the peak prevalence of stunting appeared in the year 2014, with the years 2013–2015 having the highest relative prevalence of stunting of all years. The prevalence of stunting in the years 2016–2019 was higher than the prevalence in the years 2010–2012. This makes stunting a tragic puzzle for Ethiopia, because the years 2015–2019 had relatively good economic improvements, despite the political instability across the country in the years 2016–2019.

In considering this review and meta-analysis, the authors recommend a qualitative research, mainly a focused ethnographic study to explore the culture of both rural and urban villages on caring and handling children, preparing the meals of children, health-seeking behaviour of mothers during illness, and perception of the community towards nutrition from preconception to the end of adolescence. The stunting scenario in Ethiopia contradicts with the literature that children throughout the world can attain full growth potential if they are nurtured in healthy environments and their caregivers strictly stick to the recommended health, nutrition, and health care practices [100]. However, in Ethiopia, the prevalence of stunting was not decreased, while both the economic status of the community and the health care services provided were improved. Although the economic changes brought to Ethiopia were not paramount in decreasing stunting as per the WHO 2030 plan [11] and HSTP 2015 [97], a relative reduction must occur. We are also considering the drought from 2010 to 2014 and immigration from 2016 to 2019 (Ref), which might reverse the trends of reduction in stunting. This immigration and drought effect on stunting may become doubled because of the current COVID-19 pandemic in the next years. World Health Organization is warning that COVID-19 may push millions of people into poverty and malnourishment. This is also true in Ethiopia as a number of reports indicated.

Nonetheless, the prevalence of stunting from late 2010 to 2019 should not be 41.5%. Because, unlike most of the Sub-Saharan Africa countries, Ethiopia had the highest and continuous reduction in the prevalence of stunting between 2000 and 2011, where stunting decreased from 57.7% in 2000 to 50.8% in 2005 to 44.3% in 2011 [101]. With regard to the sensitivity analysis of this review, a 43.19% (95% CI: 42.62, 43.76) prevalence of stunting was computed, while 10 papers with low scores were removed. However, the sensitivity analysis from the previous review reported a 40% (95% CI; 32, 48) prevalence of stunting [1]. Thus, the current review has a higher prevalence of stunting (by 3.19%.) The reason for such a difference in prevalence might be the studies included in our review reported a higher prevalence of stunting, particularly studies conducted from late 2016 to 2019. However, the important question is why stunting became higher than the previous review. Although the interval prevalence of the previous review and the current review is similar, there was a figurative difference. The reason might be because of the researchers concern to address the most vulnerable communities that may have not been addressed before the year 2010 and could not consider in the previous review. On the contrary, those papers might have a high prevalence of stunting and were included in our review.

The other causes for such a high prevalence of stunting in these years might be the drought in Ethiopia from 2010 to 2014 caused by La Niña [102, 103]. Currently, the number of people targeted for relief food and cash support remains largely unchanged due to the significant spike in internal displacement since April 2018 [104]. The pooled prevalence of stunting by subgroup analysis showed marked differences with regard to the prevalence of stunting among regions. According to the WHO's 2010 classification, the prevalence of stunting was considered “very high” (above 40) in the Amhara region, Ethiopia, Oromia region, Ethiopia, Tigray region, Ethiopia, and country-based studies, Ethiopia; “high” (30–39%) in the SNNP region, Ethiopia, and Afar Region, Ethiopia; and “medium” (20–29%) in Somalia Region, Ethiopia. Of the studies included in this review, there was no region that had a prevalence of stunting that was considered “low” (<20%) [44]. The high prevalence of stunting in the Amhara and Oromia regions might be as a result of many numbers of children at the household level and have the largest population in the country (EDHS, 2016), which might contribute to household food insecurity. The 2016 EDHS report [45] revealed that the prevalence of stunting was 46% in the Amhara region, 43% in the Benishangul-Gumuz region, 41% in the Afar region, 39% in the Tigray region, 39% in the SNNP region, 27% in the Somalia region, and 37% in the Oromia region. The subgroup meta-analysis reported that the prevalence of stunting was 48.21% in the Amhara region, 37.78% in the Afar region, 42.55% in the Tigray region, 36.45% in the SNNP region, 43.53% in the Oromia region, and 28.4% in the Somalia region. The 2016 EDHS report and this subgroup meta-analysis have results that agree in some regions and some results that are contradicted in other regions. For example, the subgroup analysis of this review and the 2016 EDHS report have close prevalence in the Amhara, SNNP, and Somalia regions. Regarding the associations between stunting and the wealth index, this review and meta-analysis identifies that low wealth quintiles are associated with stunting. This meta-analysis indicated that the pooled odds of stunting due to having a low/poor wealth index were 1.92 (95% CI: 1.46, 2.54) and due to having a medium wealth index was 1.33 (95% CI: 1.07, 1.65) compared to households that had a high/rich wealth index. This association of stunting and the wealth index was supported by a report that stunting is greatly influenced by three important factors: food, health, and quality of care provided for children [45].

In relation to this, other evidence has reported that combating stunting among children depends on accessing diversified and nutrient-rich foods, providing appropriate care for mothers and children, creating appropriate health care services and a healthy environment, including safe water, good hygiene, and sanitation [105]. Food, health, and health care services are affected by social, economic, and political factors. To afford the above important conditions, the socioeconomic status of a given household is an invaluable condition [106]. Although the determinant factors of stunting are diverse, socioeconomic status plays a great role in the occurrence of stunting. When socioeconomic status improves and poverty is reduced, child stunting will be improved by getting greater access to food, improved maternal and child care, and better public health care services [54, 107, 108]. This meta-analysis agreed with the study done by Krishna et al. that was conducted in four low- and middle-income countries (Ethiopia, India, Peru, and Vietnam) [109]. A further analysis of the demographic and health surveys of Rwanda (2014–2015) and Uganda (1995, 2001, 2006, and 2011) showed the same result [110] to this review. The association between stunting and wealth index was also reported by other studies conducted from south Asia (Afghanistan, Bangladesh, India, Nepal, and Pakistan) [111, 112]. Thus, socioeconomic status greatly influences the living standard, which is explained by the wealth index and in turn caused stunting. This implies that improving the living standard of the community will decrease the occurrence of stunting. The finding of this review contradicts the previous meta-analysis [56], where the wealth index was not a factor for stunting.

This disagreement might be because of differences in the sample size, search date, and number of studies included. In addition, the review by Kalkidan had considerable heterogeneity (*I*^2^ = 92%), but the heterogeneity in our review was lower (*I*^2^ = 63.8%). Thus, the lower degree of heterogeneity in our review might indicate the robustness of its scientific quality. In contrast to the previous review, we performed subgroup analyses by region and by study design. The subgroup analysis by design indicated that cross-sectional studies had moderate heterogeneity (*I*^2^ = 52.5%), but the case-control studies were homogeneous (*I*^2^ = 0%). This means that the review might be more relevant than the previous one because of its homogeneity. Additionally, the review by Kalkidan Hassen categorized the wealth index into two quintiles: low and high. However, we classified the wealth index into three quintiles: low/poor, medium, and high/rich. This might be the other reason for the contradictory pooled estimation. In contrary to the review by Kalkidan and Tefera, our review agreed with the study done by Fenske et al., where the wealth index had the largest effect on stunting [113]. The similarity might be that wealth is a universal factor for stunting irrespective of cultural, educational, and sociodemographic data, because children from low/poor wealth index households are less likely to have adequate food. This review agreed with a review that was conducted in urban setting, and reported low household income was identified as a risk factor for stunting [114, 115]. This similarity might support that the wealth index is a known underlying cause of stunting. Particularly, in urban settings, the dependence on cash flow might emphasize that household income is a real cause of stunting. The findings of the current review agree with the reports of Headey and Biadgilign who reported that the wealth index and stunting have an association [116, 117]. The similarity might be due to the fact that the study populations are similar. Both of the studies were nation-based studies on under-five children. This review agreed with a study that reported the presence of an association between stunting and wealth index, with a substantial wealth gap in stunting, even after controlling for wealth-related differences [118]. This study indicated that Ethiopian children in the top 60% of the wealth distribution are 3.9 percentage points less likely to be stunted compared with an equivalent child that is among the poorest 40% [118]. The review agreed with a nation-based study conducted in Ghana that showed the wealth index was significantly associated with stunting. The study showed that children residing in the lowest wealth quintile households had significantly increased probabilities of being stunted in comparison to children residing in the highest wealth quintile households (AOR 2.36, 95% CI: 1.29, 4.30) [119].

From this literature and our review, we recognize a reduction of stunting by 40% as per the WHO's 2030 plan might be achieved through improving the economic status of the community in collaboration with the agriculture sectors. Some authors have agreed and recommended that strengthening the existing micronutrient interventions and community-based management of severe acute malnutrition programmes [120] have public health importance in reducing the prevalence of stunting. This review is supported by another study that reported that a stunted child was more likely to have been born into a low-income household; hence, intergenerational transmission of poverty and of childhood stunting is a possibility and may become a vicious cycle [121]. This again agreed with a nation-based study in Ethiopia that reported under the recent Situational Analysis of the Nutrition Sector (SITAN), which indicated an association between poverty and stunting. The nation-based study indicated that children from lowest wealth quintile were found to be stunted [122].

This review agreed with the previous studies that reported children born to severely and moderately food insecure households were more likely to be stunted than children born to food-secure households [61, 123]. This review agreed with a study that stunting disproportionately affects children in poorer countries and from poorer households [99, 108]. The review also agreed with studies that reported poorer households experience a higher prevalence of anthropometric failure [124–128].

## 7. Conclusion

Despite the great deal of heterogeneity, the present review revealed significant findings on the pooled prevalence of stunting and its associations with wealth index, which could be used for monitoring the burden of stunting in Ethiopia. This review revealed that stunting is still stable and ominously high in the country. The burden of stunting is overriding in the Amhara region of the country, Ethiopia. Moreover, stunting is associated with the economic class in which children from low/poor economic class become stunted compared with high/rich economic classes. Thus, the implementation of policies to reverse stunting should get the concern of the government, the sector that leads Seqota Declaration, which planned to end stunting by 2020. In particular, the health and agriculture sectors need to act together to improve the socioeconomic status of the community. The intervention should consider the highly affected regions in prioritizing the intervention.

## Figures and Tables

**Table 1:** Summary of included studies in assessing prevalence and associated factors of stunting in Ethiopia, 2010–2019 [68–82].

Author	P/year	Region	Study setting	Study design	Data source	Sample size	Outcome	Reference
Abay et al.	2019	Ethiopia	Community based	Cross sectional	Primary	9495	Stunting	[63]
Abdibari et al.	2016	Somalia	Community based	Cross sectional	Primary	694	Stunting	[74]
Abel et al.	2017	Afar	Community based	Cross sectional	Primary	840	Malnutrition	[68]
Ahmed et al.	2015	Oromia	Community based	Cross sectional	Primary	453	Stunting	[59]
Amare et al.	2010	SNNP	Community based	Cross sectional	Primary	2410	Stunting	[73]
Amare et al.	2017	Amhara	Community based	Cross sectional	Primary	1295	Stunting	[80]
Amare et al	2016	Amhara	Community based	Cross sectional	Primary	681	Stunting	[84]
Araya et al.	2017	Tigray	Community based	Cross sectional	Primary	610	Malnutrition	[64]
Atanaw et.al	2018	Amhara	Community based	Cross-sectional	Primary	593	Undernutrition	[86]
Bealu et al.	2017	SNNP	Community based	Cross sectional	Primary	508	Nutritional status	[60]
Behailu et al	2014	Amhara	Community based	Cross sectional	Primary	367	Malnutrition	[76]
Behailu et al.	2014	Amhara	Community based	Cross sectional	Primary	620	Malnutrition	[76]
Birara et al.	2014	Amhara	Community based	Cross sectional	Primary	844	Stunting	[79]
Demewoz et al.	2016	NA	Community based	Cross sectional	EDHS	11,872	Stunting	[62]
Dilano	2018	Benishangul	Community based	Cross sectional	Primary	564	Stunting	[88]
Disha et al.	2013	NA	Community based	Cross sectional	Secondary	3422	Undernutrition	[61]
EDHS	2019	Ethiopia	Community based	Cross sectional	Primary	4989	Stunting	[90]
Eskeziaw et al.	2015	SNNP	Community based	Cross sectional	Primary	567	Stunting	[78]
Hiwot et al.	2017	SNNP	Community based	Cross sectional	Primary	834	Undernutrition	[75]
Hiwot et al.	2012	Oromia	Community based	Cross sectional	Primary	791	Undernutrition	[66]
Jalane	2019	Oromia	Community based	Cross sectional	Primary	616	Chronic undernutrition	[71]
Kalkidan	2017	Oromia	Community based	Cross sectional	Primary	584	Wasting &stunting	[56]
Kasahun et al.	2019	NA	Community based	Cross sectional	EDHS	8743	Stunting	[91]
Kebede et al.	2013	Oromia	Community based	Cross sectional	Primary	820	Malnutrition	[77]
Kidanemariam et al.	2016	Tigray	Community based	Case control	Primary	330	Stunting	[70]
Lamrot et al.	2018	SNNP	Community based	Cross sectional	Primary	398	Stunting	[58]
Masresha et al.	2011	SNNP	Community based	Cross sectional	Primary	575	Stunting	[67]
Misgan et al.	2016	Afar	Community based	Cross sectional	Primary	401	Stunting	[81]
Samson et al.	2019	SNNP	Community based	Cross sectional	Primary	342	Stunting	[83]
Seifu et al	2017	SNNP	Community based	Cross sectional	Primary	3975	Stunting	[55]
Selamawit et al.	2015	Amhara	Community based	Cross sectional	Primary	1287	Stunting	[26]
Shiferaw et al.	2018	Amhara	Community based	Cross sectional	Primary	410	Stunting	[42]
Taye et al.	2018	Oromia	Community based	Cohort	Primary	4468	Stunting	[87]
Terefe et al.	2017	SNNP	Community based	Case control	Primary	587	Stunting, & wasting	[82]
Tesfaye et al.	2019	Tigray	Community based	Cross sectional	Primary	394	Stunting	[69]
Wagaye et al.	2014	Amhara	Community based	Cross sectional	Primary	610	Undernutrition	[65]
Yeshalem	2017	Amhara	Community based	Cross sectional	Primary	480	Undernutrition	[89]
Yirgu et al.	2015	Somalia	Community based	Cross sectional	Primary	210	Nutritional status	[57]
Zemenu et al.	2017	Oromia	Facility based	Cross sectional	Secondary	384	Malnutrition	[25]
Zufan et al.	2019	Ethiopia	Community based	Cross-sectional	EDHS	7452	Nutritional status	[85]

*Note*. NA: not applicable; SNNPR: South Nations, Nationalities, and Peoples' Region; EDHS: Ethiopian Demographic and Health Survey.

**Table 2:** The main findings and quality assessment reports of the selected studies conducted from 2010 to 2019 about stunting in Ethiopia.

Author	Year	Male	Age	Anthropometric analysis	Confounding adjusted	Main findings	Risk (JBI)
Kalkidan	2017	289	6–24	WHO ENA	Age, residence, complementary feeding initiation, breastfeeding, dietary diversity, family size, food insecurity, wealth index, diarrhea, and farming land size	Child caring practices are independent predictors of nutritional status than wealth or economic indicators	Low
Yirgu et al.	2015	109	6–24	WHO Anthro	Maternal education, food security, age at complementary feeding, meal frequency, bottle-feeding, breastfeeding in the first 24 hours, and wealth index	Low dietary diversity scores, inappropriate age of complementary feeding initiation, and bottle-feeding were predictors of stunting	Low
Lamrot et al.	2018	171	6–59	WHO Anthro	Age, sex, birth order, maternal education, latrine availability, hand washing using soap, and ANC follow-up	41.7% of child was stunted. Age, sex, birth order, mother education, having toilet facility, and ANC follow-up associated with stunting	Low
Ahmed et al.	2015	422	24–59	WHO ENA	Confounding not adjusted	61.1% of children were stunted	High
Bealu et al.	2017	447	6–59	WHO Anthro	Food security, child sex, child age, initiation of complementary feeding, and breastfeeding status	45.6% were stunted. Household food insecurity, child age, and initiation of complementary feeding associated with stunting	Low
Disha et al.	2013	1783	6–59	NAv	Food insecurity	50.7% of children were stunted. Household food insecurity associated with stunting	High
Seifu et al	2017	1969	6–59	WHO Anthro	Age of the child, sex of the child, morbidity, place of delivery, maternal education, ethnicity/race, and household wealth index	43.7% of children were stunted. Age and sex were associated with stunting. Advanced maternal education and house hold food security were protective factors of stunting	Low
Demewoz et al.	2016	6168	6–59	NAv	Child age, sex, anemia, maternal age, maternal education, birth interval, family size, wealth index, place of residency, region, and source of drinking water	44.4% of children were stunted. Birth interval, sex of the child, sex of household head, anemia, maternal education, father's education, poverty, and maternal nutritional status	Low
Kasahun et al.	2019	4455	6–59	NAv	Child age, birth interval, wealth index, maternal education, source of drinking water, mothers body mass index, and child sex	Not breastfeeding children, children from poor households, male children, and short birth spacing associated with stunting.	Low
Araya et al.	2017	326	6–59	WHO Anthro	Mothers hand washing, cleaning material used to wash hands, source of drinking water, and age of child	36.1% of children were stunted. Age is the only factor associated with stunting	Low
Selamawit et al.	2015	622	6–59	WHO ENA	Morbidity, age of child, number of family size, marital status, father's education, and occupational status of house hold head	49.4% of children were stunted. Age of the child, number of family size, and father's educational status associated with stunting	Low
Wagaye et al.	2014	399	6–59	WHO ENA	Child age, monthly income, ANC follow-up, family size, prelacteal feeding, and maternal age at first birth	57.7% of children were stunted. Prelacteal feeding and age at first birth associated with stunting. Monthly family income was inversely associated with stunting	High
Hiwot et al.	2012	NAv	6–59	WHO Anthro	Residence, number children, age of child, birth order, mothers' BMI, and source of drinking water	45.8% of children were stunted. Number of children, age of the child, birth order, and mother's BMI associated with stunting	Low
Masresha et al.	2011	NAv	6–24	WHO Anthro	Extra food during pregnancy and lactation, prelacteal feeding, bottle-feeding, meal frequency, and dietary diversity	37.2% of children were stunted. Time of complementary food started and extra food during pregnancy and lactation associated with stunting	Low
Abel et al.	2017	476	6–59	WHO Anthro	Sex of child, age of child, time of complementary food started, child immunization status, diarrheal disease in the last 2 weeks, fever in last 2 weeks, and presence of latrine in the house	43.1% of children were stunted. Sex of child, age of the child, diarrhea in the last two weeks, and fever in the last two weeks associated with stunting	High
Tesfaye et al.	2018	172	6–59	WHO ENA	Sex of the child, marital status, mother education, mother occupation, extra food during lactation, and hand washing facility near to toilet	49.2% of children were stunted. Sex of the child and hand washing facility near to toilet associated with stunting	Low
Kidanemariam et al.	2016	164	6–24	NAv	Maternal education, mother height, birth weight, number of children under five, dietary diversity, mother BMI, repeated previous illness, age at complementary feeding, and household income	Maternal education, mother height, birth weight, number of children, dietary diversity, mother BMI, and repeated previous illness were associated factors of stunting	High
Jalane Mekonen	2019	306	6–59	WHO Anthro	Fever in the last 2 weeks, diarrhea in the last 2 weeks, age at complementary food started, duration of exclusive breastfeeding, and number of children	Stunting was associated with mother educational status, number of children, age of complementary foods started, and presence of diarrhea in the last two weeks	Low
Hiwot et al.	2017	432	6–59	WHO ENA	Age of mothers, colostrum feeding, exclusive BF in the first six months, cessation of breastfeeding status, diarrheal morbidity in the past 12 months, and sex of the child	39.3%, 15.8% and 6.3% of children were stunted, underweighted and wasted respectively. Male child, not fed on colostrums, cessation of breastfeeding before two years of age, and diarrheal morbidity in the last 12 months associated with stunting	Low
Behailu et al.	2014	330	6–59	WHO ENA	Sex of Head of HH, family size, ANC visits, child sex, domestic animals, colostrum feeding, EBF, measles sickness, latrine, protected water, birth order, presence of bed, child diarrhea, and monthly income	The prevalence of stunting, underweight, and wasting was 60.6%, 31.1%, and 12.6% in the community-based nutrition program implementing districts, respectively	High
Behailu et al.	2014	192	6–59	WHO ENA	Sex of Head of HH, family size, ANC visits, child sex, colostrum feeding, EBF, measles sickness, presence of bed, child diarrhea, and monthly income	The prevalence of stunting, underweight, and wasting was 39.0%, 27.5%, and 14.7% in noncommunity-based nutrition program implementing districts, respectively	High
Kebede et al.	2013	410	6–59	WHO ENA	Sex, age, and educational status of mothers, family monthly income, ownership of farm land, gestational age, use of family planning, and time to obtain drinking water	47.6%, 30.9%, and 16.7% of children were stunted, underweight and wasted, respectively. Child age, family monthly income, prelacteal feeding, and family planning associated with stunting	Low
Birara et al.	2014	435	6–59	WHO ENA	Sex of child, age of child in months, breastfeeding status, and wealth quintile	The prevalence of stunting, underweight, and wasting was 47.3%, 25.6%, and 8.9% (95%CI: 6.9–10.2), respectively. Age of the child, sex of the child, and breastfeed status associated with stunting	Low
Amare et al.	2017	656	6–59	WHO ENA	Wealth status, maternal education, maternal employment status, paternal education, health care access, source of drinking water, availability of latrine, breastfeeding initiation, complementary feeding initiation, and dietary diversity score	37.7% and 26.8% were moderately and severely stunted, respectively. Farming occupation of mother, lack of postnatal vitamin-A supplementation, poorer household wealth status, and accessing family food from farms were determinants of severe stunting	Low
Shiferaw et al.	2018	228	6–59	WHO ENA	Birth order, birth interval, birth weight, immunization status, diarrhea, method of feeding, age of child, duration of BF, and complementary food started	Low weight at birth, older age, mistimed initiation of complimentary feeding, and mothers' lack of ANC visit associated with stunting	Low
Misgan et al.	2016	178	6–59	WHO ENA	Sex of the child, ANC visit, minimum dietary diversity, household hunger scale, prelacteal feeding, maternal age, and monthly household income	32.2%, 23.5%, and 13.8% of children were stunted, underweight, and wasted, respectively	Low
EDHS	2019	1298	6–59	NAv	Confounding not adjusted	37% of children were stunted. The prevalence of stunting was 22% among children 6–8 months and 44% on children aged 48–59 months	Low
Abay et al.	2019	3637	6–59	NAv	Age of the child, region, mother's education, mother's BMI, wealth index, sex, size of child, and number of children	Child age, maternal education, region, wealth status, religion, sex of child, number of children, child size, water access, and toilet facility were factors of stunting	High
Amare et al	2010	974	6–59	NAv	Age of mother, sex, birth order, and family income	There is no association between malaria and undernutrition	Low
Abdibari et al.	2016	232	6–59	WHO ENA	Family size, educational status of mothers, occupations of mothers, income, child sex, and availability of latrine in the house	Factors contributing to malnutrition were immunization status, family size, child sex, monthly income, maternal education, and total duration of breastfeeding	Low
Eskeziaw et al.	2015	273	6–59	WHO Anthro	Residence, sex, age of mother, maternal education, occupational status, media exposure, place of delivery, ANC follow-up, PNC follow-up, and maternal illness	Stunting associated with child sex, ANC follow-up, maternal illness after delivery, maternal literacy, and occupation	Low
Zemenu et al	2017	80	6–59	NAv	Child age, sex, and maternal education	38.3% of children were stunted. Only maternal education was associated with stunting	High
Atanaw et.al.	2018	NAv	6–59	WHO Anthro	Mothers' occupation, number of under-five children, decision making, age of children, and wealth index	The prevalence of stunting and wasting were 42.3% and 7.3%, respectively. Poor wealth status and age of child associated with stunting.	Low
Zufan et al.	2019	3816	6–59	NAv	Sex of the child, age of the child, residence, region, family size, maternal educational status, source of drinking water, wealth index, birth order, and place of delivery	Maternal education and maternal nutritional status associated with stunting. Similarly, maternal nutritional status, place of delivery, and birth interval associated with wasting	Low
Terefe et al.	2017	569	6–24	WHO Anthro	Maternal education, wealth status, main source of family food, source of drinking water, availability of latrine, dietary diversity, and child age	The prevalence of stunting and wasting was 58.1 and 17.0%, respectively. Poor wealth status, child age of 12–24 months, and source of family food of own food production associated with stunting	Low
Samson et al.	2019	164	6–59	WHO Anthro	Child sex, child age, maternal educational status, monthly income, use of family planning, distance to obtain drinking water, family size, and prelacteal feeding	The prevalence of stunting was 24.9% with 7.9% of severely stunted. Children aged 12–23 months old, children with diarrheal morbidity, and children who received prelacteal feeding were predictors of stunting	Low
Taye et al.	2018	1419	6–59	WHO ENA	Sex of child, age of child, malaria infection, height for age, wealth status, and maternal education	44.9 of children were stunted. Children with malaria and younger age were more likely to be stunted. Stunting and wasting were not risk factors of malaria	Low
Dilano Abdisa	2018	311	6–59	WHO Anthro	Sex of child, duration of breastfeeding, family size, paternal education, and paternal occupation	32.8% of children were stunted. Family size, low dietary diversity score, duration of breastfeeding, and sex of children associated with stunting	High
Amare et al	2016	365	6–59	WHO ENA	Colostrums, family size, source of household food, and dietary diversity	The prevalence of stunting was 46%. Latrine facility and family size associated with stunting	Low
Yeshalem Mulugeta	2017	248	6–59	WHO ENA	Marital status, occupation, possession of radio, child's living situation, number of children, illness, prelacteal feeding, and initiation of complimentary feeding	The prevalence of stunting, underweight, and wasting was 42%, 22.1%, and 6.4%, respectively. Illness in the preceding two weeks, having two children under the age of three, and late initiation of complementary feeding associated with stunting	Low

*Note*. WHO ENA: World Health Organization Emergency Nutrition Assessment; WHO Anthro: WHO Anthro Survey Analyser; NAv: not available; JBI: Joanna Briggs Institute.

## Data Availability

The raw materials that support the conclusions of this systematic review are incorporated to the manuscript and presented in tables or figures in the result section and as the supplementary file.

## Abbreviations

AOR: Adjusted odds ratio
CI: Confidence interval
EDHS: Ethiopian Demography and Health Survey
UNICEF: United Nation International Children Fund
WHO: World Health Organization
WB: World Bank
SNNPR: South Nations, Nationalities, and Peoples' Region
PRISMA: Preferred Reporting Items for Systematic Reviews and Meta-Analyses
GDP: Gross domestic product
ANC: Antenatal care
BMI: Body mass index
JBI: Joanna Briggs Institute.

## Additional Points

*Limitations*. The limitation of this review was the high heterogeneity in the samples of the retrieved studies, which might be because of differences in the locations and populations of the studies. This also causes to have a pooled prevalence with high heterogeneity.

## Supplementary Materials

Supplementary Materials Supplementary Figure 1: sensitivity analysis of high-quality studies in assessing stunting among children under five years of age in Ethiopia and its 95% CI in Ethiopia, 2010–2019. Supplementary Figure 2: funnel plot to assess publication bias in assessing stunting among children under five years of age in Ethiopia, 2010–2019.
