# Supplementary material for: Low Economic Class Might Predispose Children under Five Years of Age to Stunting in Ethiopia: Updates of Systematic Review and Meta-Analysis
Source: J Nutr Metab. 2020 Dec 12;2020:2169847. doi: 10.1155/2020/2169847 (PMC7789483; doi:10.1155/2020/2169847)
Supplement: Supplementary Materials — Supplementary Figure 1: sensitivity analysis of high-quality studies in assessing stunting among children under five years of age in Ethiopia and its 95% CI in Ethiopia, 2010–2019. Supplementary Figure 2: funnel plot to assess publication bias in assessing stunting among children under five years of age in Ethiopia, 2010–2019. [file 2169847.f1.zip › 2169847.f1/Supplementary figure1.docx]

**Supplementary figure1**: *Sensitivity analysis of high quality studies in assessing stunting among children under-five years of age in Ethiopia and its 95% CI in Ethiopia, 2010-2019*
